# Machine learning approaches to predict the 1-year-after-initial-AMI survival of elderly patients

**DOI:** 10.1186/s12911-022-01854-1

**Published:** 2022-04-29

**Authors:** Jisoo Lee, Sulyun Lee, W. Nick Street, Linnea A. Polgreen

**Affiliations:** 1 Department of Business Analytics, University of Iowa, Iowa City, USA; 2 Interdisciplinary Graduate Program in Informatics, University of Iowa, Iowa City, USA; 3 Department of Pharmacy Practice and Science, University of Iowa, Iowa City, USA

**Keywords:** Acute myocardial infarction (AMI heart attack), Machine learning, Lasso logistic regression (LLR), Random forest (RF), Sampling methods, Hyper-parameter optimization, Nested cross-validation (CV)

## Abstract

**Background:**

While multiple randomized controlled trials (RCTs) are available, their results may not be generalizable to older, unhealthier or less-adherent patients. Observational data can be used to predict outcomes and evaluate treatments; however, exactly which strategy should be used to analyze the outcomes of treatment using observational data is currently unclear. This study aimed to determine the most accurate machine learning technique to predict 1-year-after-initial-acute-myocardial-infarction (AMI) survival of elderly patients and to identify the association of angiotensin-converting- enzyme inhibitors and angiotensin-receptor blockers (ACEi/ARBs) with survival.

**Methods:**

We built a cohort of 124,031 Medicare beneficiaries who experienced an AMI in 2007 or 2008. For analytical purposes, all variables were categorized into nine different groups: ACEi/ARB use, demographics, cardiac events, comorbidities, complications, procedures, medications, insurance, and healthcare utilization. Our outcome of interest was 1-year-post-AMI survival. To solve this classification task, we used lasso logistic regression (LLR) and random forest (RF), and compared their performance depending on category selection, sampling methods, and hyper-parameter selection. Nested 10-fold cross-validation was implemented to obtain an unbiased estimate of performance evaluation. We used the area under the receiver operating curve (AUC) as our primary measure for evaluating the performance of predictive algorithms.

**Results:**

LLR consistently showed best AUC results throughout the experiments, closely followed by RF. The best prediction was yielded with LLR based on the combination of demographics, comorbidities, procedures, and utilization. The coefficients from the final LLR model showed that AMI patients with many comorbidities, older ages, or living in a low-income area have a higher risk of mortality 1-year after an AMI. In addition, treating the AMI patients with ACEi/ARBs increases the 1-year-after-initial-AMI survival rate of the patients.

**Conclusions:**

Given the many features we examined, ACEi/ARBs were associated with increased 1-year survival among elderly patients after an AMI. We found LLR to be the best-performing model over RF to predict 1-year survival after an AMI. LLR greatly improved the generalization of the model by feature selection, which implicitly indicates the association between AMI-related variables and survival can be defined by a relatively simple model with a small number of features. Some comorbidities were associated with a greater risk of mortality, such as heart failure and chronic kidney disease, but others were associated with survival such as hypertension, hyperlipidemia, and diabetes. In addition, patients who live in urban areas and areas with large numbers of immigrants have a higher probability of survival. Machine learning methods are helpful to determine outcomes when RCT results are not available.

## Background

Acute myocardial infarction (AMI), commonly known as a heart attack, is a life-threatening condition in which blood flow to the heart is abruptly blocked, causing damage or death of the heart muscle. According to Benjamin et al. [1], the overall prevalence of AMIs in adults over 20 was 7.9 million (3 percent) in the United States. Each year, about 790,000 adults aged over 35 in the US experience AMIs. Unfortunately, readmission and mortality are not uncommon in the years following the initial AMI. Of the people who experience AMI in a given year, 1 in every 4 has recurrent AMIs, and 1 in 7 results in death [1].

In order to reduce morbidity and mortality after the first AMI and prevent subsequent AMIs, multiple randomized controlled trials (RCTs) have determined the relative value of different interventions to prevent or treat AMIs and to provide guidance for patients [2–9]. However, the results of RCTs do not always apply to all groups of patients and this absence is a particular problem for elderly patients with AMIs. The average age of the first AMI is approximately 65 years for males and 72 years for females [1]. RCTs may exclude patients for various reasons including age, specific comorbidities, or other health conditions. Thus, appropriate recommendations for treatment may not be evident because the results may not generalize to patients who do not meet the trial’s enrollment criteria.

In such cases, observational data are often used to make data-driven decisions about treatment. Applying machine learning to retrospective data can aid clinicians by identifying high-risk patients and understanding the factors that lead to that risk. Yet, to our knowledge, no studies have investigated exactly which machine learning technique is likely to be most accurate to analyze the risk of AMIs in elderly patients.

The objective of this study is to determine the most accurate machine learning technique to predict 1-year-post-AMI survival of elderly patients, and to identify the types of predictive variables that lead to the most accurate predictions. Among all predictive variables, we are specifically interested in the association of angiotensin-converting enzyme inhibitors (ACEis) and Angiotensin II Receptor Blockers (ARBs), specific hypertension treatments that are recommended post AMI [10], with the elderly AMI patients’ survival. We examined the performance of widely used machine learning techniques: lasso logistic regression (LLR) and random forest (RF).

## Methods

### Study cohort

In order to build data models to predict 1-year-post-AMI survival of elderly patients, we used a cohort of 124,031 Medicare beneficiaries who experienced an AMI (an inpatient stay with the primary diagnosis code 410.x1) in 2007 or 2008. The Chronic Condition Data Warehouse provided all Medicare claim information (e.g., providers, diagnoses, and procedures), enrollment information (e.g., demographics), and part D prescriptions (specific information about each prescription).

We considered only Medicare beneficiaries who have complete information for one year before and after the index date or until the date of death. The index date was defined as the admission date of inpatient stay for AMI. To ensure data completeness, a Medicare beneficiary with AMI was included if he or she (1) was 66 years or older at the index date; (2) did not have an AMI in the year prior to the index date; (3) was discharged alive from the index stay and survived for at least 30 days after the index stay; (4) did not use hospice or skilled nursing care for the 30 days after the index stay; (5) had Medicare part A (hospitalization coverage) and part B (medical insurance) for the entire year prior to the index date; (6) had Medicare part D (prescription drug coverage) for the 6 months prior to the index date; (7) had Medicare parts A, B, and D for either the entire one year after the index date or until the date of death. Moreover, if a patient had multiple AMIs in 2007 or 2008, then only the record relevant to his or her first AMI was included in the dataset.

In an effort to control for potential confounders, we included a wide range of covariates; for example, patient demographics; socioeconomic characteristics of the patient’s residential area from the 2000 U.S. Census (based on a postal code) (e.g., low income area, high poverty area, etc.); medical conditions (cardiac events and comorbidities) during the pre-index period and the index stay; medications taken for the 180 days before the index date and post-AMI; complications; procedures; insurance (e.g., benefit phase); and the use of different facilities during the index stay.

Further, we adjusted some of these variables in an attempt to examine their impacts in various approaches. For example, summary measures of comorbidities (e.g., the total number of comorbidities, Charlson Comorbidity Index (CCI, from CCW), and Elixhauser Comorbidity Index (ECI (see Additional file 1))) were measured to check the burden of disease mix for the pre-index and index periods. For these variables, the change between the two periods (index stay score minus pre-index score) was also calculated. In the case of dual eligibility, when the patient was eligible for both Medicare and Medicaid, because Medicaid eligibility changes, we recorded how it had changed over the periods (e.g., dual eligible for both periods, only eligible for the pre-index period, only eligible for the index period, and ineligible for both periods).

ACEi/ARB use was defined as a filled prescription for either an ACEi or an ARB in the 30 days after the index date to perform analysis “on an intention-to-treat basis” [7].

For the purpose of analysis, all variables were categorized into nine different groups: ACEi/ARB use, demographics (age, gender, race, etc.), cardiac events (cardiac arrest, arrhythmia, stroke, etc. across different time frames), comorbidities (myopathy, angioedema, hyperlipidemia, etc. across different time frames), complications (cardiogenic shock, sepsis, and pneumonia during the index period), procedures (cardiac catheterization, stent, etc. across different time frames), medications (diuretics, beta blockers, etc. during the pre-index period), insurance (cumulative beneficiary responsibility amount, cumulative total cost, etc. during either index or pre-index period), and utilization (acute inpatient stay, post-acute care, etc. during the index period). The list of all variables in each category can be found in more detail (see Additional file 2).

The institutional review board of the University of Iowa approved this study.

### Model design

Our outcome of interest was 1-year-post AMI survival (to be precise, mortality). This binary dependent variable was recorded as 1 if a patient died within one year after the index date of AMI and 0 otherwise.

For our classification task, we experimented with (1) logistic regression and (2) random forest (RF) approaches. Logistic regression was chosen as a commonly used model for linear fitting. RF was selected to take into account the possible complex interactions among features with non-linear relationships. More information about these two algorithms is provided later in this paper.

We used the area under the receiver operating characteristics (ROC) curve, or simply AUC as a measure for assessing the performance of predictive algorithms. AUC is considered the better performance evaluation metric than other widely used ones, such as accuracy and the Matthews correlation coefficient (MCC), to effectively evaluate and compare classification models over imbalanced datasets as in our case [11]. However, we also considered other performance evaluation metrics including accuracy, sensitivity, and specificity.

The dataset was imbalanced with total 19,418 out of 124,031 elderly patients (15.66%) who died within one year after the index AMI. To deal with this class imbalance, we compared three common strategies: under-sampling, over-sampling, and a combination of the two (both-sampling). In under-sampling, records from the majority class (0, survived) were randomly removed. In contrast, in over-sampling, records from the minority class (1, died) were duplicated to provide a balanced dataset. Both-sampling is a mix of under-sampling and over-sampling to balance the majority and minority.

We took different approaches to category and variable selection: forward selection for category selection and backward elimination for feature selection. In the case of category selection, we started to build a model with a single category and then kept adding other categories one by one. We continued until adding more categories did not significantly increase prediction performance. On the other hand, we applied backward elimination for variable selection for RF. This is an iterative method that starts with all features and removes the least significant variable that enhances the performance until no enhancement is observed. Since LLR includes the feature selection procedure in its optimization function, we did not use backward elimination for LLR. Specifically, LLR performs L1 regularization to shrink the coefficients towards zero and eliminate comparatively insignificant variables from the model [12].

Nested 10-fold cross-validation (CV) was performed to create an unbiased estimate of AUC as well as to handle feature and hyper-parameter selection. Hyper-parameter selection is the procedure for optimizing the set of parameters used in machine learning models, so that they can yield the best performing predictions [13]. Hyper-parameters that we estimated here are lambda for LLR; the number of variables available for splitting at each tree node, and the number of trees to grow for RF. A detailed explanation for each hyper-parameter will be provided in the following section. In the inner loop, we selected a subset of categories and different combination of variables within each category and a value of hyper-parameters that enhanced AUC of the inner loop the most. Note that the feature selection was treated as an extension of the hyper-parameter optimization problem, so we used the same inner AUC for the optimization of both features and hyper-parameters. In the outer loop, the model was trained with the selected category/variable subset and hyper-parameters and was used to estimate the AUC in the outer loop.

**Fig. 1:**
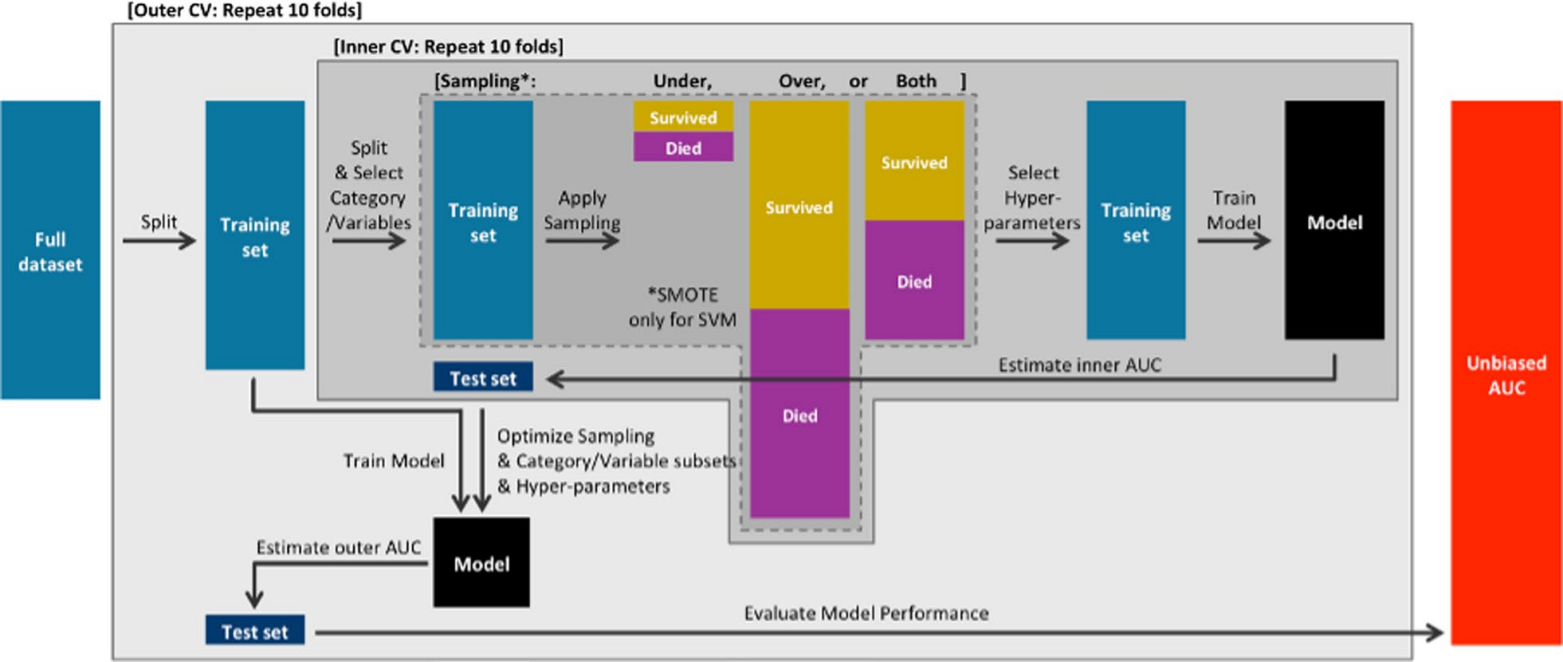
Schematic of model development for survival prediction. We optimized feature and hyper-parameter selection in the inner CV loop, while we evaluated the model performance with the optimal feature subsets and hyper-parameters in the outer CV loop. Both inner and outer layers consist of ten repeated folds (training/testing repetitions)

Figure 1 illustrates the entire process of model development. First, the dataset was partitioned into 10 subsets (folds) of nearly equal size with roughly equal proportions of target patients. In the outer loop, while holding out one fold as a test set, the rest were assigned as a training set. Likewise, in the inner loop, the training set was split and assigned to another test set and training set. Then, the sampling method was applied to the inner training set. We extracted a subset of categories/variables and tried different values of the hyper-parameters to train the model and compare its performance as estimated with the inner test set. The feature subset and hyper-parameter with the highest inner AUC were selected for each inner loop. Repeating the same process throughout the inner 10 folds, the most frequently appearing (or the averaged) feature subsets and hyper-parameters were finally chosen to train a model with the outer training set. The model performance was evaluated by averaging the outer AUC.

All analysis was performed using R statistical software version 3.5.0 and Python version 3.7.1.

### Algorithms

#### Lasso logistic regression (LLR)

Logistic regression is a widely used model when the dependent variable is binary, $y \in \{0,1\}$. Unlike linear regression, its goal is to model the probability *p* that the output variable *Y* takes on 0 or 1 given the input variables $x \in \{x_1, x_2, \dots x_n\}$. It can be expressed mathematically as:

1 $$\begin{aligned} Pr(y|x) = p^{y}(1-p)^{1-y}. \end{aligned}$$

By applying the logistic function, it can be converted into:

2 $$\begin{aligned} Pr(y|x;\beta ) = \frac{1}{1+e^{-(\beta ^{T}x)}}^y \left(1-\frac{1}{1+e^{-(\beta ^{T}x)}} \right)^{1-y}. \end{aligned}$$

With a large number of predictors, reducing the coefficients of less predictive variables along with fitting the parameters of the model is vital not only for prediction accuracy but also for model interpretability. To fit the parameter, the principal of maximum log-likelihood is often applied, which maximizes the product of probabilities.

3 $$\begin{aligned} \log L(\beta ) = \sum _{i=1}^n y\beta ^{T}x - \log \Big (1 + e^{\beta ^{T}x}\Big ) \end{aligned}$$

LLR adds an L1-norm penalty term to the likelihood optimization [14]. The complexity is controlled with the parameter $\lambda$.

4 $$\begin{aligned} \widehat{\hat{\beta }}(\lambda ) = \min _{\beta } \left[ -\frac{1}{n} \sum _{i=1}^n y\beta ^{T}x - \log \Big (1 + e^{\beta ^{T}x}\Big ) +\lambda \left\Vert \beta \right\Vert _1\right] \end{aligned}$$

As $\lambda$ increases, this penalty forces the coefficients to shrink toward zero. In this way, Lasso regularizes and selects variables. To fit the regularization parameter lambda $\lambda$, we performed cross-validation (grid search) to find a value of $\lambda$ at the minimum of mean squared error.

#### Random forest (RF)

Random forest (RF) is an ensemble classification technique, where multiple decision trees are constructed on random samples of features in order to boost accuracy and protect against overfitting [15].

A decision tree is a top-down logical tree that splits the given samples based on the value of a chosen feature that can divide the sample into homogeneous groups. Its fundamental limitation is high variance in model prediction. Trees tend to overfit the training data as they grow in complexity. In RF, this limitation is solved by employing an ensemble method called bootstrap aggregation (bagging), where each tree is built using randomly drawn samples from the original data and each tree gives an independent vote for its predicted class label [16, 17]. Although any individual tree in the ensemble may be sensitive to the training set and thus inaccurate, the final majority vote across numerous trees greatly reduces the variance and is often remarkably accurate [16].

We tuned two hyper parameters of RF, the level of randomness (*mtry*) and the size of the forest (*ntree*). The former is decided by number of variables to be examined at each split. A lower value generates less correlated trees, so it leads to more stable but potentially less accurate performance. The latter is controlled by the number of trees in the model. A higher value can achieve improved performance but increases the computation time linearly [13].

## Results

The results of the two algorithms, LLR and RF , built with each individual feature category are summarized in Tables 1 and 2 respectively, and the bold text indicates the selected category subset in each selection step with the highest AUC. As discussed, we chose the average value of the parameter or the most frequently selected value of the parameter with the highest inner AUC for optimization and reported the average of outer AUCs. In the table, the category subset used, the best sampling method, the optimum value of parameters, and the corresponding outer AUC value and its standard deviation are presented.

**Table 1:** Lasso logistic regression with one category subset

One category subset	Sampling	Lambda	AUC (SD)
Demographics	Under	0.000655	0.6798 (0.0077)
Cardiac events	Both	0.001200	0.6359 (0.0086)
**Comorbidities**	**Both**	**0.000595**	**0.7531 (0.0053)**
Complications	Under	0.002252	0.5822 (0.0067)
Procedures	Under	0.000900	0.7241 (0.0043)
Medications	Under	0.001153	0.6369 (0.0055)
Insurance	Both	0.000453	0.6196 (0.0064)
Utilization	Under	0.001875	0.6243 (0.0065)

The AUC and the SD columns show the average of outer AUC and its standard deviation respectively. The most commonly selected sampling method and the average of lambda were reported under the Sampling and the Lamda columns as well

**Table 2:** Random forest with one category subset

One category subset	Sampling	mtry	ntree	AUC (SD)
Demographics	Under	3	250	0.6695 (0.0059)
Cardiac Events	Under	3	2250	0.6327 (0.0093)
**Comorbidities**	**Under**	**3**	**2750**	**0.7459 (0.0045)**
Complications	Under	3	1500	0.5804 (0.0069)
Procedures	Under	3	1250	0.7183 (0.0061)
Medications	Under	3	1500	0.6317 (0.0055)
Insurance	Under	3	1250	0.6127 (0.0068)
Utilization	Under	3	2500	0.6224 (0.0055)

The AUC and the SD columns show the average of outer AUC and its standard deviation respectively. The most frequently selected sampling method and parameters (mtry and ntree) are reported accordingly

LLR and RF showed comparable performance across all category subsets, with the performance of LLR slightly better than that of RF. Both of these models generated the best result with the comorbidity-category subset (AUC = 0.7531 for LLR and 0.7459 for RF).

By adding different categories one by one to the selected subset with the best AUC result, the experiments continued until the increase of AUC was not significant. The results are presented in Tables 3, 4, 5, 6, 7 and 8. The best AUC performance was achieved with the same combination of category subsets (comorbidities, procedures, demographics, and utilization) for both LLR and RF, but with different sampling methods, both-sampling for LLR and under-sampling for RF.

**Table 3:** Lasso logistic regression with two category subset

Two category subset	Sampling	Lambda	AUC (SD)
Comorbidities + demographics	Both	0.000508	0.7785 (0.0049)
Comorbidities + cardiac events	Both	0.000665	0.7568 (0.0054)
Comorbidities + complications	Both	0.000581	0.7537 (0.0054)
**Comorbidities + procedures**	**Both**	**0.000489**	**0.7849 (0.0039)**
Comorbidities + medications	Both	0.000543	0.7553 (0.0053)
Comorbidities + insurance	Both	0.000385	0.7563 (0.0056)
Comorbidities + utilization	Both	0.000567	0.7573 (0.0053)

**Table 4:** Random forest with two category subset

Two category subset	Sampling	mtry	ntree	AUC (SD)
Comorbidities + demographics	Under	3	750	0.7705 (0.0044)
Comorbidities + cardiac events	Under	3	750	0.7503 (0.0049)
Comorbidities + complications	Under	3	1250	0.7467 (0.0046)
**Comorbidities + procedures**	**Under**	**3**	**2500**	**0.7804 (0.0039)**
Comorbidities + medications	Under	3	2000	0.7502 (0.0045)
Comorbidities + insurance	Under	3	1750	0.7501 (0.0044)
Comorbidities + utilization	Under	3	1000	0.7512 (0.0049)

**Table 5:** Lasso logistic regression with three category subset

Three category subset	Sampling	Lambda	AUC (SD)
**Comorbidities + procedures + demographics**	**Under**	**0.000489**	**0.7942 (0.0035)**
Comorbidities + procedures + cardiac events	Both	0.000565	0.7851 (0.0039)
Comorbidities + procedures + complications	Both	0.000481	0.7850 (0.0040)
Comorbidities + procedures + medications	Both	0.000467	0.7860 (0.0040)
Comorbidities + procedures + insurance	Both	0.000362	0.7860 (0.0039)
Comorbidities + procedures + utilization	Both	0.000480	0.7862 (0.0040)

**Table 6:** Random forest with three category subset

Three category subset	Sampling	mtry	ntree	AUC (SD)
**Comorbidities + procedures + demographics**	**Under**	**3**	**2000**	**0.7894 (0.0035)**
Comorbidities + procedures + cardiac events	Under	3	500	0.7810 (0.0038)
Comorbidities + procedurse + complications	Under	3	2000	0.7804 (0.0036)
Comorbidities + procedures + medications	Under	3	2000	0.7829 (0.0039)
Comorbidities + procedures + insurance	Under	3	1500	0.7827 (0.0038)
Comorbidities + procedures + utilization	Under	3	2750	0.7825 (0.0040)

**Table 7:** Lasso logistic regression with four category subset

Four category subset	Sampling	Lambda	AUC (SD)
Comorbidities + procedures + demographics + cardiac events	Under	0.000527	0.7946 (0.0036)
Comorbidities + procedures + demographics + complications	Under	0.000466	0.7944 (0.0036)
Comorbidities + procedures + demographics + medications	Under	0.000919	0.7948 (0.0035)
Comorbidities + procedures + demographics + insurance	Both	0.000409	0.7949 (0.0034)
**Comorbidities + procedures + demographics** **+ Utilization**	**Both**	**0.000441**	**0.7955 (0.0036)**

**Table 8:** Random forest with four category subset

Four category subset	Sampling	mtry	ntree	AUC (SD)
Comorbidities + procedures + demographics + cardiac events	Under	6	1750	0.7902 (0.0040)
Comorbidities + procedures + demographics + complications	Under	3	2000	0.7898 (0.0038)
Comorbidities + procedures + demographics + medications	Under	6	2750	0.7901 (0.0031)
Comorbidities + procedures + demographics + insurance	Under	6	3000	0.7907 (0.0036)
**Comorbidities + procedures + demographics + utilization**	**Under**	**6**	**2750**	**0.7911 (0.0037)**

For potential clinical utility, in addition to AUC, the performance of LLR and RF with the best corresponding settings of category subset, sampling method, and hyper parameters was further evaluated using other supplementary metrics (accuracy, sensitivity, and specificity). The cross-validated values are reported in Table 9. Nevertheless, those values can be changed by simply modifying a predictive threshold (in this study, it is 0.5). LLR showed outstanding classification performance in terms of not only AUC but also sensitivity. When studying survival, models with high levels of sensitivity are preferable.

**Table 9:** Performance evaluation with final category subset (comorbidities + procedure + demographics + utilization)

Model	Sampling	AUC (SD)	Accuracy (SD)	Sensitivity (SD)	Specificity (SD)
LLR	Both	**0.7955 (0.0036)**	0.7104 (0.0039)	**0.7490 (0.0076)**	0.7033 (0.0054)
RF	Under	0.7911 (0.0037)	**0.7890 (0.0043)**	0.5322 (0.0090)	**0.8367 (0.0051)**

Due to its superior performance as determined by AUC, LLR with the final category subset (comorbidities, procedures, demographics, and utilization) and both-sampling method was chosen as a final model. Again, note that we used AUC as a base performance evaluation metric to deal with the class imbalance problem. Table 10 shows the coefficients of the variables from the final model. The variables with zero coefficients were excluded by the LLR as the feature selection proceeded. Variables from the final model are cross-tabulated with survival (see Additional file 3).

**Table 10:** Coefficients of features selected by final model

Category	Features	Time periods	Coef.
Intercept	Intercept	–	$-1.1207$
ACEi/ARBs use	ACEi/ARBs (untreated)	Post-index	0.1910
Comorbidities	Charlson comorbidity index (CCI)	Index	0.1102
	Charlson comorbidity index (CCI)	Pre-index	0.0932
	Elixhauser comorbidity index (ECI)	Pre-index	0.0420
	Elixhauser comorbidity index (ECI)	Index	0.0165
	Number of comorbidities	Pre-index	0.0027
	Number of comorbidities	Index	0.0005
	Charlson comorbidity index (CCI)	Change	0.0000
	Elixhauser comorbidity index (ECI)	Change	0.0000
	Number of comorbidities	Change	0.0000
	Serious myopathy	Pre-index	0.5637
	General cancer	Index	0.4801
	Heart failure	Index	0.3594
	Metastatic cancer	Pre-index	0.2553
	Metastatic cancer	Index	0.2453
	Heart failure	Pre-index	0.2102
	Atrial fibrillation	Index	0.1367
	Serious myopathy	Index	0.1205
	COPD	Pre-index	0.1183
	Hypotension	Pre-index	0.1108
	Depression	Pre-index	0.1020
	Chronic kidney disease	Index	0.0882
	COPD	Index	0.0842
	Hyperkalemia	Pre-index	0.0786
	Atrial fibrillation	Pre-index	0.0671
	Hepatic events	Index	0.0572
	Hyperkalemia	Index	0.0488
	Depression	Index	0.0464
	Renal failure	Pre-index	0.0264
	Non-AMI ischemic heart disease	Pre-index	0.0233
	Hepatic events	Pre-index	0.0115
	Renal failure	Index	0.0083
	Chronic kidney disease	Pre-index	0.0072
	Hypotension	Index	$-0.0028$
	Non-AMI ischemic heart disease	Index	$-0.0076$
	Bradycardia	Pre-index	$-0.0086$
	Hypertension (uncomplicated)	Pre-index	$-0.0255$
	General cancer	Pre-index	$-0.0515$
	Hypertension (complicated)	Index	$-0.0608$
	Diabetes	Pre-index	$-0.0624$
	Hypertension (complicated)	Pre-index	$-0.0954$
	Non-serious myopathy	Index	$-0.1136$
	Bradycardia	Index	$-0.1146$
	Hypertension (uncomplicated)	Index	$-0.1274$
	Diabetes	Index	$-0.1290$
	Non-serious myopathy	Pre-index	$-0.1372$
	Asthma	Index	$-0.1573$
	Hyperlipidemia	Index	$-0.2294$
	Asthma	Pre-index	$-0.2325$
	Angioedema	Index	$-0.2511$
	Hyperlipidemia	Pre-index	$-0.2883$
	Angioedema	Pre-index	$-0.4396$
Procedures	Echocardiography	Index	0.3932
	Percutaneous coronary intervention	Index	0.3223
	Stent	Index	0.2612
	Stent	Pre-index	0.1310
	Pacemaker implantation	Pre-index	$-0.0330$
	CABG	Pre-index	$-0.2485$
	Pacemaker implantation	Index	$-0.2629$
	Stress test	Index	$-0.3152$
	Cardiac catheterization	Index	$-0.5627$
	CABG	Index	$-0.9250$
Demographics	Age: 85+	–	0.9877
	Age: 81–85	–	0.5803
	Age: 76–80	–	0.3469
	Age: 71–75	–	0.1136
	Metro area: unknown	–	0.7015
	Metro area: non-metro	–	0.0183
	Dual eligibility	Steady [2]	0.3618
	Dual eligibility	Steady [1]	0.0004
	Dual eligibility	Steady [3]	0.0000
	Dual eligibility	Index	0.2281
	Dual eligibility	Pre-index	0.1213
	Low income subsidy	–	0.1231
	Low income area	–	0.0445
	Low high school diploma area	–	0.0279
	High poverty area	–	$-0.0398$
	High immigrant area	–	$-0.0456$
	No English speaker area	–	$-0.0469$
	Race: black	–	0.0386
	Race: white	–	$-0.0096$
	Race: unknown	–	$-0.0853$
	Race: Asian	–	$-0.1035$
	Race: hispanic	–	$-0.1974$
	Race: others	–	$-0.3169$
	Average life expectancy: 4th quartile	–	$-0.0513$
	Average life expectancy: 2nd quartile	–	$-0.0589$
	Average life expectancy: 3rd quartile	–	$-0.0681$
	Gender: male	–	$-0.1724$
Utilization	ER use	Index	0.1240
	Acute inpatient stay days	Index	0.0215
	Post-acute care use	Index	0.0001
	Transferred to another facility	Index	$-0.1429$

Lasso with Four-Category Subset—Comorbidities, Procedures, Demographics, and Utilization—, and Both Sampling (LLR coefficients of the variables from the final model with four categories)

The result of the final model illustrates that the more comorbidities the patient has, the lower the survival rate with AMI; CCI both on and prior to the index admission date most clearly shows this association. Regardless of the diagnosis date, having serious myopathy, heart failure, metastatic cancer, atrial fibrillation, depression, COPD, CKD, hyperkalemia, hepatic events, or renal failure have critical impacts on the mortality of AMI patients. Meanwhile, those with angioedema, hyperlipidemia, asthma, non-serious myopathy, diabetes, hypertension, and bradycardia were more likely to survive after one year of initial AMI.

Regarding procedures, having echocardiography, percutaneous coronary intervention, or a stent on the day of the AMI is associated with mortality. On the other hand, having pacemaker implantation, CABG, a stress test, or cardiac catheterization was associated with survival.

Some demographic characteristics also influence post-AMI mortality. For example, patients with older ages or who received a low-income subsidy are at greater risk of death within a year after AMI. Likewise, the patients who had been consistently eligible for both Medicare and Medicaid have a lower survival rate. The patient’s dual eligibility during the index period is more strongly linked to mortality than the dual eligibility during the pre-index period. In addition, black or female patients have lower survival rates than males or patients of other races.

The characteristics of the patient’s residential area are another set of factors. The mortality risk increases if the patient lives in an area with a low income rate, low high school diploma rate, or in the first quartile of average life expectancy. However, the survival probability increases when the patient lives in a metro area, an area with high poverty or high immigrant rates, or a high portion of non-English speakers.

Using the emergency room or post-acute care, as well as increased inpatient length of stay are associated with a lower probability of survival, but patients who are transferred to another facility have higher probability of survival.

Lastly, ACEi/ARBs use is associated with survival. If an elderly AMI patient does not fill a prescription of either ACE or ARB in the 30 days after AMI, the risk of mortality increases.

## Discussion

In this paper, we used machine-learning methods to predict survival in post-AMI Medicare beneficiaries. We found that ACEi/ARB use is associated with 1-year survival for elderly patients who have suffered an AMI.

RCTs are the gold standard for studying treatment effectiveness [18]. However, because RCTs are expensive, time consuming, and often exclude elderly patients and those with comorbidities, treatment effectiveness needs to be determined for excluded patients in other ways. Using insurance claims data and machine-learning methods are an alternative solution to determine treatment effectiveness when RCTs are difficult or impossible to perform. In fact, the Food and Drug Administration, which has relied on RCT data in the past, is now interested in obtaining “Real-World Evidence” from “Real-World Data” including electronic medical records, insurance claims and data obtained directly from patients [19].

However, unlike RCTs where data analysis is usually straightforward, when using real-world data, determining the optimal data-analysis method is often difficult. In this paper, we examined two different machine-learning methods, LLR and RF. We found that LLR was better than RF for predicting the 1-year-after-initial-AMI survival of patients.

Interestingly, a similar conclusion, that regularized logistic regression models perform better than RF models when predicting survival after an AMI, was reported in [20–22]. In [20], ridge logistic regression with binarized features resulted in the best 10-fold validated AUC at 0.832 among all models (decision tree, naive bayes, artificial neural network, etc.). This study differs from ours in several aspects, such as the prediction of 30-day AMI mortality and the limited number of patients (603) and attributes (23, mostly from blood tests). In [21], logistic model trees and simple logistic algorithms with all combined categories of dataset (demographics, admission, lab and chart, treatment, and diagnostic information) resulted in the best 10-fold validated AUC at 0.901 among all models, while RF with the same dataset scored 0.893. It intended to predict long-term (1-year) mortality, but used a relatively small set of variables (79) and patients (5436). Lee et. al [22] reported that penalized (Lasso and Ridge) logistic regression generally performs the best for predicting short and long-term (3 and 12 months) survival of patients with ST-segment elevation myocardial infarction (STEMI) and non-ST-segment elevation myocardial infarction (NSTEMI). The study involved 14,183 adult patients in Korea and a wide range of their characteristics (demographics, past medical history, initial symptoms, lab findings, events before ED arrival and during the hospital stay, and coronary angiographic findings).

The outstanding performance of LLR is mainly due to its penalized effect with a Lasso regularization term, which reduces the variability of model by shrinking the coefficients of unnecessary features toward or possibly to zero and selecting only necessary features [23]. It helps LLR to have better predictive ability for datasets where the number of features is far greater than the number of samples, such as our case. In addition, the fact that the linear model was more effective for predicting our particular outcome indicates that a linear combination of features provides substantial information about the outcome while nonlinear models add respectively little marginal predictive value. In other words, this implies that the patterns and relationships between variables related to the 1-year-after-initial-AMI survival of elderly patients can be drawn by a relatively simple linear model with considerably fewer features. In addition to predictive performance, the interpretability of RF is very limited compared to LLR. The association and importance of variables can be evaluated by using the estimated coefficients of LLR [24], providing a meaningful and easy-to-understand interpretation of results both for clinicians to determine who is most at risk and researchers to begin further studies with appropriately automatic feature selection.

In addition to the effectiveness of ACEi/ARBs for post-AMI treatment among Medicare beneficiaries, we have other interesting results. Many of our results are not surprising. For example, patients who have a higher comorbidity burden, represented by a higher CCI, have a lower probability of survival. In addition, patients with serious comorbidities with high mortality rates, such as heart failure, metastatic cancer and CKD also have a lower probability of survival after AMI. Also, as has been found previously, poverty is associated with a lower probability of survival; those who are eligible for Medicaid or live in a low-income area or an area with lower educational attainment, have a lower probability of survival [25].

However, there were some surprising results. For example, patients with hyperlipidemia, diabetes and hypertension have an increased probability of survival. Although most comorbidities are generally associated with lower rates of guideline treatment [26], in a previous study, we found that patients with comorbidities, especially diabetes, were more likely to fill a prescription for at least one guideline-recommended post-AMI treatment than those without comorbidities [27]. The increased survival among patients with specific comorbidities may be related to increased rates of guideline-recommended treatment.

We also found that patients who live in urban areas, areas with large numbers of immigrants and non-English speakers also have increased probability of survival. Although many immigrants may have healthier lifestyles than native-born populations, one would expect that they would also have decreased access to healthcare. Indeed, previous findings have been mixed. One study in Israel found that mortality after AMI was increased for immigrants compared to native-born patients [28]. However, another study from Denmark found that, in general, immigrants had lower mortality after an AMI than native-born patients [29]. The effect of immigration on AMI survival should be examined in further work.

Our study has several limitations. First, considering medications, we do not know if patients are actually taking the medications. We only know that they filled a prescription for the medication. Second, some of our features are ecological. For example, not all patients living in a low-income area are low income. Third, all diagnoses, procedures and comorbidities were determined using diagnostic codes, and some codes have better sensitivity and specificity than others. Fourth, despite our efforts to include a wide range of covariates, there may be other factors that are not included in this study but affect the patient’s survival, for instance, consecutive AMIs or other excluded cardiac events. Fifth, ACEi/ARB use might be associated with outcomes other than survival, and these will be considered in future work. Last, but not least, the machine learning models provide an association between features and outcomes, but do not necessarily imply causation. Therefore, our models cannot support causal relationships with survival.

In conclusion, we found that LLR is an effective method for predicting the 1-year-after-initial-AMI survival of elderly patients. In addition, ACEi/ARBs are associated with patient survival among our cohort of post-AMI older adults, many with significant comorbidities.

## Supplementary Information

**Additional file 1**. The table illustrates how the ICD-9-CM codes of comorbidities for ECI are converted into those for research variables.

**Additional file 2**. The table contains the list of variables used for 1-year-after-AMI survival of elderly patients in this paper.

**Additional file 3**. The table shows the correlations between variables, such as the mortality and survival rate of patients aged 66-70 years with or without ACEi/ARBs treatment, extracted from descriptive analysis.

## Data Availability

The data that support the findings of this study are available from ResDAC (resdac.org), but restrictions apply to the availability of these data, which were used under license for the current study, and so are not publicly available. Please contact Linnea Polgreen (corresponding author) with questions.

## Abbreviations

ACEi: Angiotensin-converting enzyme inhibitor
AMI: Acute myocardial infarction
ARB: Angiotensin II receptor blockers
AUC: Area under the curve
CCI: Charlson comorbidity index
CCW: Chronic condition data warehouse
CKD: Chronic kidney disease
COPD: Chronic obstructive pulmonary disease
CV: Cross-validation
ECI: Elixhauser comorbidity index
LLR: Lasso logistic regression
RBF: Radial basis function
RCT: Randomized controlled trial
RF: Random forest
ROC: Receiver operating characteristics
